# Combating Combination of Hypertension and Diabetes in Different Rat Models

**DOI:** 10.3390/ph3040916

**Published:** 2010-03-26

**Authors:** Talma Rosenthal, Firas Younis, Ariela Alter

**Affiliations:** Department of Physiology and Pharmacology, Sackler School of Medicine, Tel Aviv University, Israel; E-Mails: firasyou@post.tau.ac.il (F.Y.); alterariela@gmail.com (A.A.)

**Keywords:** hypertensive-diabetic animal models – treatment, Zucker rats, Goto-Kakizaki rats, SHROB rats, SHR/NDmcr-cp rats, Cohen Rosenthal diabetic hypertensive rats

## Abstract

Rat experimental models are used extensively for studying physiological mechanisms and treatments of hypertension and diabetes co-existence. Each one of these conditions is a major risk factor for cardiovascular disease (CVD), and the combination of the two conditions is a potent enhancer of CVD. Five major animal models that advanced our understanding of the mechanisms and therapeutic approaches in humans are discussed in this review: Zucker, Goto-Kakizaki, SHROB, SHR/NDmcr-cp and Cohen Rosenthal diabetic hypertensive (CRDH) rats. The use of various drugs, such as angiotensin-converting enzyme (ACE) inhibitors (ACEIs), various angiotensin receptor blockers (ARBs), and calcium channel blockers (CCBs), to combat the effects of concomitant pathologies on the combination of diabetes and hypertension, as well as the non-pharmacological approach are reviewed in detail for each rat model. Results from experiments on these models indicate that classical factors contributing to the pathology of hypertension and diabetes combination—Including hypertension, hyperglycemia, hyperinsulinemia and hyperlipidemia—can now be treated, although these treatments do not completely prevent renal complications. Animal studies have focused on several mechanisms involved in hypertension/diabetes that remain to be translated into clinical medicine, including hypoxia, oxidative stress, and advanced glycation. Several target molecules have been identified that need to be incorporated into a treatment modality. The challenge continues to be the identification and interpretation of the clinical evidence from the animal models and their application to human treatment.

## 1. Introduction

Experimental hypertensive type I diabetes can be easily produced by injecting streptozotocin to spontaneous hypertensive rats (SHR), and this model is used extensively for studying physiological mechanisms [1] and treatments [2,3,4,5,6]. Streptozotein-induced diabetes in Dahl’s rate is also a good model for insulin-dependent diabetic hypertensive rat. However, this model is rarely used in contrast to SHR-type I diabetes [7,8]. Type 2 diabetes is much more common and hypertension occurs two times more frequently in diabetic than in nondiabetic individuals. The addition of hypertension aggravates the complications of diabetes, which by itself is a major independent risk factor for cardiovascular disease (CVD). Indeed, the co-existence of the two conditions [9,10] is a powerful promoter of CVD, accelerating microvascular and macrovascular complications and greatly increasing cardiovascular stroke and end stage renal disease risk [11]. The ideal treatment for this combined pathology includes ACEIs, various ARBs, and CCBs. However, since different mechanisms are responsible for the two pathologies and response rate to treatments is far from homogenous and ideal, the search for additional therapeutic agents continues. Advances in our understanding of the mechanisms and therapeutic approaches in humans have depended on a variety of animal models and because there are no reliable mouse models on hypertension, the focus is on rat models, including the following:

1) Zucker rats, which develop moderate hypertension only while becoming obese. This is an ideal model of progressive nephropathy.
2) Goto-Kakizaki rats, in which hyperglycemia is not associated with overt proteinuria or progressive nephropathy. This salt-sensitive strain will develop hypertension when fed a high salt diet or given Na-retaining agents.
3) SHROB rats – Koletsky rats, which are obese, hyperinsulinemic and hypertriglyceridemic, and exhibit proteinuria. Despite extreme pathology, hypertension is not exacerbated compared to SHR.
4) SHR/NDmcr-cp rats – a substrain of the SHR/N-cp rat, which has a genetic background from the SHR and carries nonsense mutation of leptin receptor derived from the obese Koletsky rat.
5) Cohen Rosenthal diabetic hypertensive nonobese rat model, a result of cross-breeding SHR and Cohen diabetic rats (CDR).

The use of various drugs to combat the effects of concomitant pathologies of the combination of diabetes and hypertension as well as the non-pharmacological approach will be reviewed. The results of studies with the various treatments are summarized in Table 1.

**Table 1:** Pharmacological and non-pharmacological treatments studied in hypertensive/diabetic animal models.

Animal model	Treatment	Main results	Reference
**Zucker fatty rats**	Pharmacological		
	ARB derivativesR-147176	strongly inhibited advanced glycation while less effective than olmesartan in AT1R binding, it minimally lowers blood pressure Significant renoprotection.	Izuhara *et al*., 2008 [72]
	Nateglinide insulinotropic agent + Telmisartan	Improved glucose metabolism Restored lowered plasma adiponectin levels	Kajioka *et al*.,2007 [26]
	Losartan	Lowered blood pressure No significant effect on albuminuria, or glomerular or tubulointerstitial injury	Crary *et al*., 1995 [19]
	Losartan	Improved both early and late survival of large MI Reduced adrenergic stimulation accompanied by fewer ventricular arrhythmias	Pourdjabbar *et al*., 2005 [21]
	Irbesartan	Preserved renal function and metabolic profile Substantially improved survival	Janiak *et al*., 2006 [22]
	Olmesartan	Slowed progression of nephropathy in type 2 diabetes without affecting glucose metabolism.	Mizuno *et al*., 2006 [23]
	Candesartan *versus* perindopril	Both induced RAS blockade, slowing the progression of glomerulosclerosis, and preserving glomerular cells Both suppressed proteinuria.	Sebekova *et al*., 2009 [24]
	Losartan and Ramipril; Vasopeptidase inhibitor AVE7688.	Improved diabetic nephropathy by RAS inhibition on several levels, unrelated to its effects on blood pressure and glycemic control, by renal oxidative stress-dependent mechanisms. Reduced renal AGE formation in type 2 diabetes more effectively than the blockade of RAS	Portero-Otín *et al*., 2008 [32]
	Lovastatin, a cholesterol synthesis inhibitor	Reduced glomerular injury, leaving glomerular area or glomerular macrophage content unchanged	O'Donnell *et al*., 1993 [29]
	Enalapril + HMG-CoA reductase inhibitor – statin	Attenuated endothelial-dependent responses in coronary vessels of both Zucker Obese and ZDF rats.	Oltman *et al*., 2008 [31]
	Non- pharmacological		
	Various combinations of essential oils	Fenugreek may block glucose absorption Cinnamon may have insulin-like action and affect insulin signaling	Talpur *et al*.,2005 [34]
	Stevia rebaudianabertoni (SrB)	SrB extracts lowered plasma glucose in diabetics Stevioside + soy protein SPI exhibited preventive action on development of type 2 diabetes	Jeppesen *et al*., 2006 [46]
	Quercetin, a flavonoid abundant in fruits and vegetables	Reduced blood pressure Prevented morphological and functional changes in heart, vessels and kidney	Perez-Vizcaino *et al*., 2009 [36]
Goto-Kakizaki rats	Pharmacological		
	Omapatrilat and Enalapril	Comparable blood pressure-lowering and renoprotective properties Omapatrilat prevented vascular dysfunction in diabetes more effectively than enalapril	Cheng *et al*., 2005 [39]
	Non- pharmacological		
	Diterpene glycoside stevioside (SVS) and soy bean protein	Combination has positive synergistic effects on components of metabolic syndrome: hypertension, hyperglycemia, dyslipidemia	Jeppesen *et al*., 2006 [46]
	Lupin and soy protein	Lupin improved endothelium-dependent vasorelaxation	Pilvi *et al*., 2006 [47]
	Cereal fiber barley	Significantly reduced systolic blood pressure lowered plasma levels of total cholesterol, triacylglycerol, and LDL	Li *et al*., 2004 [48]
SHR/ND mcr-cp rats	Pharmacological		
	TelmisartanAmlodipineMoxonidine, selective I imidazdin receptor agonist	All three significantly lowered blood pressure Only telmisartan improved impaired relaxation in response to acetylcholine and the increased protein expression of endothelium NO synthase in thoracic aortas	Kagota *et al*., 2007 [62]
	Telmisartan	Prevented impaired vasorelaxation Reduced sGC expression Raised nitrotyrosine content in mesenteric arteries	Kagota *et al*., 2009 [63]
	Caloric restriction Olmesartan NifedipinePioglitazoneCobalt	Caloric restriction corrects metabolic abnormalities and protects kidney without correcting hypertension ARB and CCB lower blood pressure to the same extent, but only ARBs protect the kidney without changes in metabolic abnormalities Proglitazone provides renoprotection unlike insulin Cobalt protects kidney without correcting hypertension and metabolic abnormalities Renoprotection almost always associated with decreased AGE formation	As reviewed in Miyata *et al*., 2008 [64]; and Miyata & van Ypersele de Strihou, 2009 [85]
	Cobalt	Did not correct hypertension and metabolic abnormalities in hypertensives Reduced proteinuria and histological kidney injury, attributed to up-regulation of HIF and HIF-regulated genes and to alleviation of advanced glycation and oxidative stress	Ohtomo *et al*., 2008 [66]
	Valsartan	Improved renoprotection at doses higher than required for maximal effect on blood pressure.	Tominaga *et al*., 2009 [60]
	Hydralazine and Olmesartan	Both agents improved functional and morphologic renal damage, associated with decreased accumulation of AGE in the kidney.	Nangaku *et al*., 2003 [69]
	Olmesartan (among others) + Hydralazine	Both similarly lowered blood pressure Olmesartan significantly improved all biochemical and molecular parameters related to glomerular and tubulointerstitial damage Hydralazine relieved renal damage but less effectively than olmesartan	Watanabe *et al*., 2009 [70]
	R-147176 + Olmesartan	R-147176 induced significant renoprotection R-147176 minimally reduced blood pressure R-147176 strongly inhibited advanced glycation R-147176 bound AT1R less effectively than olmesartan	Yasui *et al.,* 2007 [75]
	Non- pharmacological (Natural)		
	Fiber-supplemented diet	Prevented abnormalities in the metabolic syndrome much more effectively than an insoluble diet	Yasui *et al*., 2007 [73]
SHROB rats			
	Captopril and S-allylmercaptocapto-pril (CPSSA) = pharmacological and nonpharm-acological approach	Reduced multiple abnormalities of metabolic syndrome. Allylmercaptocaptopril improved glucose tolerance, lowered blood pressure, reduced cardiac hypertrophy, protected against renal disease, and prevented weight gain.	Ernsberger *et al*., 2007 [58]
CRDH rats	Pharmacological		
	Omapatrilat	Beneficial effect on glycemic control	Hofman & Rosenthal, 2004 [78]
	Lercanidipine	Beneficial effect on pathology of myocardium and coronary arteries	Amenta *et al*., 2003 [79]
	Lercanidipine	Prevented changes in small-sized arteries and glomerular arterioles	Rosenthal *et al*., 2007 [80]
	Telmisartan and Valsartan	In addition to its hypotensive effect, only telmisartan demonstrated beneficial thiazolidinedione-like effects	Younis *et al*., in press [82]
	CPSSA	Prevention of weight gain, hypotensive and hypoglycemic	Younis *et al*., in press [83]

## 2. Animal Models

### 2.1. Zucker Rats

A genetic model of the metabolic syndrome is supplied by the obese Zucker rat, featuring simultaneous occurrence of obesity, hyperglycemia, hyperinsulinemia, hypertriglyceridemia, hypercholesterolemia, and moderate hypertension, bringing the model in line with patients having non-insulin dependent diabetes mellitus (NIDDM, type 2) and accompanying hypertension. Lean Zucker rats provide appropriate controls for the obese rat; however, while their inbreeding yields more consistent results, they may be less representative of a heterogeneous population. Zucker obese rats share several features with obese humans with insulin-resistant type II diabetes, including a strong genetic contribution to the transmission of obesity. Opinion differs, however, on whether the obese Zucker rat is sufficiently hypertensive compared to lean rats. According to Alonso-Galicia *et al*. [12], conflicting results could stem from different measurement techniques, ages and/or genders of the rats, and factors like amount of sodium intake. In their study, the Zucker obese rats had higher mean arterial pressure than lean Zucker rats and this rise in blood pressure was partially dependent on the renin-angiotensin system (RAS); this was evidenced by the greater reduction in blood pressure in these animals compared to lean rats during chronic Ang II blockade with losartan. Obese Zucker rats develop moderate hypertension in contrast to lean Zucker rats.

According to Osmond *et al*. [13], the smaller lumen diameter of these obese animals and larger infarction also indicates that Zucker fatty rats have significant hypertension. Infarction was 58% larger after ischemia in these animals compared to the lean ones. 

According to McCaleb and Sredy (14), all obese Zucker rats had increased urinary albumin excretion which was dependent on age and independent of hyperglycemia and glucosuria. These obese animals showed significantly higher plasma levels of glucose, insulin, total cholesterol and triglycerides than the lean Zucker rats [15]. The obese rats also showed elevated glucose and albumin concentrations in the urine compared to the lean rats. 

Studies have been conducted on numerous types of drugs aimed at combating the effects of combined diabetes and hypertension in this model.

Izuhara *et al*. [16,17] attributes the benefits of ARBs to inhibition of advanced glycation end products (AGE) and oxidative stress inherent to their chemical structure, and not necessarily to a blood pressure lowering effect and angiotensin II type 1 receptor AT1R affinity. R-147176, a compound of the family of synthesized ARB derivatives, proved to be a powerful inhibitor of AGE. When this compound was examined for AT1R affinity and pharmacokinetic parameters, it strongly inhibited advanced glycation but was 6,700 times less effective than olmesartan in AT1R binding. Its ability to lower blood pressure was minimal, but it gave significant renoprotection in three experimental rat models with renal injury, which highlights its added value: Zucker diabetic fatty rat, type 2 diabetic obese rats (SHR/NDmcr-cp); and remnant kidney rats.

TRC4186, an AGE-breaker that has been shown both *in vitro* and *in vivo* to decrease the burden of AGEs, was found to preserve cardiac function and reduce severity of renal dysfunction [18]. 

The impact of losartan on glomerular and tubulointerstitial injury was examined in obese Zucker rats [19]. While blood pressure fell, the drug did not significantly affect albuminuria or glomerular or tubulointerstitial injury. The authors concluded that in the chronic renal failure in these rats without increased intrarenal RAS activity, angiotensin II (AII) receptor antagonist may not be nephroprotective despite a reduction in blood pressure. However, it is important to note that RAS activation was reported in Zucker diabetic fatty rats: augmented angiotensinogen together with intrarenal oxidative stress are predecessors of renal injury in Zucker diabetic fatty rats [20]. Thus, the RAS cannot be ignored.

In a study by Pourdjabbar *et al.* [21], losartan improved both early and late survival of large myocardial infarction by reducing adrenergic stimulation with an accompanying reduction in ventricular arrhythmias.

The hypertension in Zucker fatty rats was thought to be related in part to the enhanced sympathetic activity observed in these animals [12]. 

Treatment of obese Zucker rats for 13 months with irbesartan preserved renal function and metabolic profile, substantially improving survival [22]. The drug lowered elevated urinary protein excretion, plasma creatinine and urea nitrogen levels, and reduced the extent of glomerular and tubulo-interstitial lesions together with a reduction of urinary monocyte chemoattractant protein-1 (MCP-1) excretion. Irbesartan also averted the rise in plasma total cholesterol, triglycerides and glucose levels, and partially corrected LDL/HDL cholesterol ratio. 

Mizuno *et al.* [23] found that olmesartan slowed the progression of nephropathy associated with type 2 diabetes without affecting glucose metabolism, adding evidence (at least partial) of independence of this renal protective effect from the antihypertensive action of the drug. The drug suppressed rises in blood urea and increased the survival rate of the Zucker diabetic fatty rats, in which histological examination disclosed its beneficial effect on renal damage. Olmesartan had a positive effect on the glomeruli and tubulointerstitium of the Zucker diabetic fatty rat kidneys, which were lessened by the drug as evidenced by an increase in the macrophage infiltration and MCP-1 expression.

Study was made of the ability of the ACEI perindopril and the ARB candesartan to reverse the established renal injury in Zucker diabetic fatty rats, which were uninephrectomized and fed a high-protein diet [24]. Both treatments inducing RAS blockade retarded the progression of glomerulosclerosis and preserved glomerular cells in this study. Both treatments suppressed proteinuria. Candesartan halted and perindopril induced a limited regression of mesangiolysis. Tubulointerstitial and vascular sclerosis scores were not significantly affected. The missing effect on the extraglomerular structures may reflect the persisting risk factors of renal damage, e.g., hyperglycemia, hypertension, dysplipidemia, obesity, and high protein intake. 

In a study in humans, candesartan either lowered or induced mild changes in plasma lipids [25]. Perindopril and candesartan both effectively lowered blood pressure in this group of patients with mild hypertension and type 2 diabetes. Perindopril improved some metabolic parameters compared with candesartan. However, the inclusion/exclusion criteria in this study could limit extrapolating the results to a general population [25].

A combination of telmisartan, and nateglinide, a rapid-onset/short-duration insulinotropic agent, for the treatment of postprandial hyperglycemia and metabolic derangements in Zucker fatty rats was studied by Kajioka *et al*. [26]. Postprandial hyperglycemia was ameliorated in these rats fed twice daily, and 6 weeks of the combination reduced fasting plasma insulin, triglycerides, and free fatty acid levels, and improved the responses of blood glucose to insulin and lowered the decremental glucose areas under the curve in the rats. Co-administration of antihypertensive drugs has the potential of management of cardiovascular risk factors, and is rapidly gaining attention that may lead to the development of combination drugs [27].

In view of reports that the formation of AGEs is reduced by both ACE inhibitors and AT_1_ receptor blockers, it appears that AVE7688 has potent chelating activity and reduces Nε-carboxymethyl-lysine (CML) formation by inhibiting metal-catalyzed formation of AGE compounds. According to these results, vasopeptidase inhibition appears to more effectively reduce renal AGEs formation in type 2 diabetes than the blockade of RAS – an effect that is significantly connected to the reduction of diabetic nephropathy [28]. 

Improved AGE clearance and direct inhibition of AGE formation by chelation may contribute to reduced accumulation of renal AGEs and to the nephroprotective effects of vasopeptidase inhibition in type 2 diabetes. 

Lovastatin, a cholesterol synthesis inhibitor, injected daily to obese Zucker rats with established nephropathy for 18 weeks reduced glomerular injury without changing either glomerular area or glomerular macrophage content [29]. Mesangial cells cultured from glomeruli of 26-week-old proteinuric obese Zucker rats on lovastatin displayed a significant dose-dependent inhibition of serum-stimulated mesangial cell DNA synthesis, indicating a slowing of the progression of established glomerular disease in these rats, and indeed high dose atorvastatin resulted in humans in reduction of blood pressure independently to lipid lowering effect changes [30].

Rosuvastatin, an HMG-CoA reductase inhibitor, or enalapril were tested for capacity to ameliorate vascular dysfunction associated with the metabolic syndrome and type 2 diabetes [31]. This study was undertaken in view of the lipid lowering action and antioxidant activity of HMG-CoA reductase inhibitor (statin). Zucker obese rats reacted with attenuation of endothelial-dependent responses in coronary vessels compared to response in lean rats. Oxidative stress, and vascular dysfunction were improved in obese fatty Zucker rats. 

In view of the increasing oxidative stress resulting from the deleterious action of the RAS on diabetic nephropathy, three RAS inhibitors were studied in the kidneys of Zucker rats: ramipril, losartan, and the vasopeptidase inhibitor AVE7688 [32]. In this study on the protein modifications induced by oxidative and carbonyl stress, 2,4-dinitrophenylhydrazine (DNP), glutamic semialdehyde (GSA), and lipoxidation-[Nε-(malondialdehyde)-lysine-(MDAL)] levels rose in all obese rats, and were decreased by AVE7688 in a dose-dependent manner, but less effectively by ramipril and losartan. Thus, diabetic nephropathy is improved by RAS inhibition on several levels (not associated with its effects on blood pressure and glycemic control) by renal oxidative stress-dependent mechanisms.

The reduction in albuminuria and morphological damage was also not impressive with ramipril in a study by Schafer *et al.* [33], whereas AVE7688 almost completely prevented albuminuria in Zucker diabetic fatty rats in addition to greatly reducing the incidence and severity of glomerulosclerosis and tubulointerstitial damage.

#### 2.1.1. Nonpharmacological Approach – Natural Treatments

Various combinations of essential oils such as fenugreek, cinnamon, cumin and oregano have been tested for their ability to enhance insulin sensitivity. Fenugreek may act by blockage of glucose absorption while cinnamon was reported to have insulin-like action and affect insulin signaling. Cumin, pumpkin seed and oregano had little effect, although data from animal studies suggest that cumin may lower circulating lipid levels in diabetic rats [34].

Guarani Indians in Paraguay and Brazil use the plant *Stevia rebaudiana Bertoni* (SrB) in their traditional treatment of diabetes. Oral intake of SrB extracts did indeed suppress plasma glucose in diabetic subjects in a long-term trial with stevioside combined with soy protein SPI. The regimen demonstrated preventive action on the development of type 2 diabetes and the metabolic syndrome in the Zucker diabetic fatty rat: glucose level decreased and the blood lipid profile improved [35].

Quercetin, one of the abundant flavonoids present in fruits and vegetables, and probably the most widely studied flavonoid because of its high biological activity, reduced blood pressure in rat models of metabolic syndrome, including obese Zucker rats and rats treated with a high-sucrose, high-fat diet. It also prevented morphological and functional changes in the heart, vessels and kidney, and the reactive oxygen species production associated with hypertension was increased [36]. 

### 2.2. Goto-kakizaki Rats

Several generations of repeated inbreeding of glucose-intolerant Wistar resulted in the Goto-Kakizaki (GK) rat, a moderately diabetic rat strain. This strain does not display hyperlipidemia or obesity, in contrast to several other rodent models of non-insulin-dependent diabetes. Structural changes related to age are associated with hyperglycemia in this rat – changes like those seen in patients with prolonged NIDDM who have not developed overt renal disease [37]. However, neither overt proteinuria nor progressive nephropathy accompanies the hyperglycemia in this model. In fact, the hypertensive GK rat is ideal for studies on the mechanisms of diabetic nephropathy, since the condition occurs in long-standing type 2 diabetes only when secondary injurious mechanisms such as hypertension are present [38]. 

Studies on the spontaneously diabetic GK rat demonstrated that hypertension and diabetes-induced vascular and renal complications were exacerbated by a high-sodium diet [39]. The latter conditions were ameliorated by omapatrilat and enalapril, which also normalized blood pressure and albuminuria during a normal-sodium diet. Greater endothelium-dependent relaxation to acetylcholine was seen with omapatrilat than with enalapril. These findings led the authors to conclude that while the two drugs have comparable blood pressure-lowering and renoprotective properties, vascular dysfunction during diabetes is more effectively prevented by omapatrilat than by enalapril in GK rats. 

Administration of deoxycorticosterone acetate (DOCA) salt to GK and Wistar rats for 24 weeks [38] resulted in enhanced macrophage infiltration into the kidney and increased renal immunohistochemical staining for MCP-1; it also induced an impressive increase in proteinuria in the hypertensive GK rats from 12 weeks. These animals also showed increased tubulointerstitial damage compared to hypertensive Wistar rats. 

Olearczyk and co-workers [40] created an AII infusion model of hypertension by continuous infusion of AII *via* an Alzet osmotic mini-pump, and a high-salt diet containing 8% (w/v) NaCl. These rats exhibited a 17-fold increase in urinary albumin excretion, which could be lowered by 2 weeks of treatment with 12-(3-adamantan-1-yl-ureido)-dodecanoic acid (AUDA), cannabinoid – a soluble epoxide hydrolase inhibitor, that resulted in decreased glomerular and tubular damage. AUDA treatment was also found to ease macrophage infiltration and inhibit urinary excretion of MCP-1 and kidney cortex MCP-1 gene expression. The authors suggest that in hypertensive GK type 2 diabetic rats, the kidney benefits, at least in part, by inhibition of the inflammatory component of nephropathy. 

When the effects of AUDA on renal injury and the infiltration of pro-inflammatory cells into the kidney were studied in another experiment from the same group, AUDA significantly attenuated an increase of pro-inflammatory cells into the kidney cortex. That mean arterial pressure was unaffected by oral administration of AUDA in hypertensive GK rats was interesting in light of reports that the administration of AUDA and other sEH inhibitors decreased blood pressure in other animal models of hypertension. While higher doses of AUDA might be required to affect blood pressure, as stated by the authors themselves, AUDA did have positive effects on the hypertensive GK rats. Thus, the results point to AUDA’s renal protective effects as occurring by a mechanism other than the one that acts on blood pressure.

In view of the 50% increase in albuminuria in salt-sensitive hypertension, Cheng and co-workers [41] studied this factor in GK rats. They found that GK rats with salt-sensitive hypertension exhibited increased monocyte/macrophage infiltration into the kidney accompanied by increased immunostaining for intracellular adhesion molecule-1 (ICAM-1). They concluded that hypertension in GK rats is salt sensitive and is associated with endothelial dysfunction and perivascular inflammation. AT(1) receptor blockade with valsartan administered to animals on a low-sodium diet improved inflammation parameters and gave partial protection against salt-induced vascular damage by blood pressure-independent mechanisms. 

Morphology and contractile response and endothelial function of resistance arteries from GK rats were studied by Brondum *et al.* [42]. Exposure to the maximal noradrenalin (NE) concentration caused significantly more tension in arteries from GK rats compared to control Wistar (CW) rats, demonstrating that the diabetic GK rat’s mesenteric small arteries have increased contractile response to NE, normal endothelial function, and unaltered morphology.

Several studies have proposed an inflammatory process by which local cytokine/chemokine production and immune cell infiltration regulate islet dysfunction and insulin resistance in type 2 diabetes. Ehses and co-workers [43] examined tissue inflammation in the GK rat, centering their attention on the pancreatic islet and the role of Interleukin-1 (IL-1). They found elevated islet IL-1beta activity in the GK rat that increases cytokine and chemokine expression and leads to the recruitment of innate immune cells. They propose that IL-1beta is not directly cytotoxic, but rather may drive tissue inflammation that affects both beta cell functional mass and insulin sensitivity in type 2 diabetes.

IL-1Ra injected subcutaneously twice daily for 4 weeks reduced islet caspase-1 mRNA by 40% and IL-1b mRNA by 50% compared to more than 90% reductions in tumor necrosis factor alpha (TNFα) and chemokine mRNAs. The pattern emerging from these data is that increased islet TNFα and chemokines are mainly IL-1 driven, while caspase-1, IL-1b, and IL-6 are partly increased in an IL-1-independent manner in the GK rat.

Ehses’ group [43] reported that IL-1Ra treatment improved hyperglycemia in GK animals by improving both beta cell insulin processing (reducing the proinsulin/insulin ratio) and insulin sensitivity; and that the reduced hyperglycemia was paralleled by reduced islet inflammation and anti-inflammatory effects on the liver.

#### 2.2.1. Nonpharmacological Approach

Jeppesen and co-workers [44] showed that diterpene glycoside stevioside (SVS) has a dual positive effect, antihypertensive and hypoglycemic, which appears to act via a calcium antagonist mechanism, like verapamil [45]. Jeppesen’s group [46] later showed that in GK rats, the combination of SVS and soy bean protein, each of which benefits diabetes, has positive synergistic effects on hyperglycemia, hypertension and dyslipidemia.

Pilvi *et al.* [47] compared the effects of lupin and soy protein on hypertension and vascular functions in GK rats and found that the both substances normalized the decreased vasocontraction in the NaCl-fed control group, while the impaired endothelium-dependent vasorelaxation was ameliorated only by lupin treatment. These findings indicate that the improvement in hypertension may be due to the corrected vascular dysfunction.

Li *et al.* [48] found that a diet of cereal fiber barley significantly decreased systolic blood pressure from week 12 and lowered the plasma levels of total cholesterol, triacylglycerol, and low-density lipoprotein cholesterol. Indeed, plasma lipids levels and systolic blood pressure were significantly positively correlated. 

### 2.3. Koletsky-SHROB Rats

Several studies have been carried out on the SHROB model, considered to be useful for investigating the interactions of metabolic abnormalities that constitute Syndrome X, including genetic obesity, genetically determined hypertension, and hyperinsulinemia, hypertriglyceridemia and renal disease with proteinuria [49,50,51,52,53,54]. Ernsberger and co-workers [55] noted that the numerous metabolic disorders and extreme insulin resistance do not enhance hypertension in these animals compared to SHR, indicating that insulin resistance and hypertension are independent in this model.

The weight of mature SHROB rats peaks at 750–1000 g, with obese males slightly but not significantly heavier than females at all ages. SHROB rats display severe proteinuria by 6 months of age, having begun as early as 6 weeks of age and accelerating exponentially [52,53]. 

Speculating that increased sympathetic nervous system activity may contribute by separate pathways to hypertension and to insulin resistance, Friedman *et al.* [56] and Ernsberger *et al.* [57] from the Koletsky group examined the chronic effects of sympathetic inhibition with moxonidine on glucose metabolism. In addition to lowering blood pressure, the drug also ameliorated glucose intolerance. According to their findings, this animal model of obese hypertension demonstrated that chronic inhibition of sympathetic activity with moxonidine therapy can reduce free fatty acids and significantly improve insulin secretion, glucose disposal, and expression of key insulin signaling intermediates.

In a joint study of The Weizmann Institute and Tel Aviv University, both captopril and allylmercaptocaptopril, a conjugate of captopril with allicin which is an active principle in garlic, were shown to be effective in attenuating multiple abnormalities of metabolic syndrome. Allylmercaptocaptopril improved glucose tolerance, lowered blood pressure, reduced cardiac hypertrophy, protected against renal disease, and prevented weight gain. Since these animals are very obese and the compound prevents weight gain, the Koletsky-SHROB appears to be a good model for proving the advantage of this unique compound [58].

### 2.4. SHR/NDmer-cp Rats

The SHR/NDmcr-cp rat is considered a very suitable animal model of the metabolic syndrome. An animal model of metabolic syndrome was established by Hirakoa-Yamamoto and co-workers [59]. This sub-strain of the SHR/N-cp rat has a genetic background from the spontaneously hypertensive rat (SHR) and a nonsense mutation of leptin receptor derived from the obese Koletsky rat. Compared to a control group of age-matched normotensive animals, the Wistar-Kyoto rat (WKY), the SHR/NDmcr-cp rat develops severe hypertension, with systolic blood pressure climbing by age 12 weeks as high as 200 mmHg [59]. Obese SHR/NDmcr-cp, which are homozygous for the leptin receptor mutation, are associated with hyperphagia that leads to obesity with metabolic syndrome and eventually to nephropathy. This rat also exhibits a number of metabolic disorders, including hyperglycemia, hyperinsulinemia, and hyperlipidemia; and histology of tissues discloses islet area expansion, fatty liver and glomerulosis. Like patients with metabolic syndrome, these rats have been reported to have increased oxidative stress [60].

According to Nagase [60], this model exhibits enhanced aldosterone signaling podocyte injury and proteinuria, which are ameliorated by epleronone or tempol. 

In a study of three drugs – amlodipine, telmisartan and moxonidine – Kagota *et al.* [62] found that all three significantly lowered blood pressure, but only telmisartan ameliorated the impairment of relaxation in response to acetylcholine and the increased protein expression of endothelium NO synthase in thoracic aortas. Endothelium-dependent relaxation decreased in the thoracic aortas of SHR/ND-mcr-cp rats despite increased nitric oxide (NO) production from the endothelium. Telmisartan lowered the serum levels of lipid peroxide and 8-hydroxy-2'-deoxyguanosine, oxidative stress markers, and the aortic levels of the protein expression of gp91, a component of NADPH oxidase, and 3-nitrotyrosine, a biomarker of peroxynitrite. These findings suggest that NADPH oxidase-derived superoxide, probably produced due to stimulation of AT1 receptors, reacts with NO to form peroxynitrite and consequently decreases active NO, leading to attenuation of endothelium-dependent relaxation. 

In isolated mesenteric arteries of male 18-week-old SHR/ND-mcr-cp, relaxations in response to acetylcholine and sodium nitroprusside were impaired and this impaired relaxation was not restored by treatment with NADPH-oxidase inhibitor apocynin [63]. Administration of telmisartan prevented the impaired vasorelaxation, decreased sGC expression, and increased nitrotyrosine content in mesenteric arteries. 

The SHR/NDmcr-cp also exhibits intrarenal AGE accumulation and its reduction may reflect a decreased oxidative stress [64]. ARBs and CCBs lowered blood pressure to the same extent, but only ARBs protected the kidney without changes in metabolic abnormalities. Insulin controled glycemia better than pioglitazone, but the latter, unlike insulin, provided renoprotection, perhaps due to the up-regulation of transforming growth factor beta (TGFβ) by hyperinsulinemia. 

A number of therapeutic modalities have been employed to bring about renoprotection as evidenced by the status of albuminuria and histology [64]. These include caloric restriction [65], anti-hypertensive agents, ARB (olmesartan), CCB (nifedipine), beta blocking agents (atenolol), lipid-(bezafibrate) or glucose-lowering agents (insulin and pioglitazone), and cobalt chloride. Renoprotection was not necessarily associated with blood pressure or glycemic control but was associated with reduced AGE formation in most studies, with the exception of insulin, which induces hyperinsulinemia that eventually leads to overproduction of transforming growth factor beta. AGE formation was reduced in two ways: directly by *in vitro* active compounds (e.g., ARBs) or indirectly by *in vitro* inactive compounds (e.g., pioglitazone and cobalt). The reduction in the later cases may be due to decreased oxidative stress, evidenced by the concomitant marked reduction in oxidative stress markers [64]. 

Cobalt is a key contributor to the defense against hypoxia by virtue of its enhancement of the activity of the hypoxia-inducible factor (HIF) [66]. While it did not correct hypertension and metabolic abormalities in hypertensive, type 2 diabetic rats with nephropathy, it did reduce proteinuria and histological kidney injury – evidence that it achieves renal protection independently of metabolic status and blood pressure. This renal protection was attributed to the up-regulation of HIF and HIF-regulated genes and to alleviation of advanced glycation and oxidative stress. Cobalt – an essential mineral and an integral part of vitamin B12 – is considered to be toxic, although it is found in very small amounts in some foods, including black tea [67] and dried fruits [68].

At doses of valsartan higher than 120 mg/kg per day, no further lowering of blood pressure was observed [60], pointing to the maximum effect afforded by 80–120 mg/kg. Higher doses of valsartan did, however, further lower proteinuria.

A comparative study of hydralazine and olmesartan [69] assessed glomerular lesions and kidney pentoside content after 20 weeks. Olmesartan reduced blood pressure and kidney pentoside content and improved histological proteinuria and renal damage. Hydralazine had a similar effect but reached statistical significance only for kidney pentoside content.

A comparison of the effects of hydralazine and ARB on hypertension and renal function showed that olmesartan significantly improved all biochemical and molecular parameters related to glomerular and tubulointerstitial damage, and hydralazine alleviated renal damage but less effectively than olmesartan, even though they showed similar lowering of blood pressure. Glucose and lipid metabolism did not differ significantly in vehicle-treated, hydralazine-treated and ARB-treated SHR/NDmcr-cp animals [70]. Findings point to the strong contribution of the RAS to the pathogenesis of renal damage in metabolic syndrome, and the powerful renoprotection conferred by ARBs to patients with metabolic syndrome.

Despite minimal reduction in blood pressure, R-147176 offered significant renoprotection in rat models with renal injury [16,70] in SHR/NDmcr-cp Zucker diabetic fatty rats, and in remnant kidney rats. It was a powerful inhibitor of advanced glycation but was far less effective than olmesartan in AT1R binding. The compound is orally bioavailable and toxicologically safe. It seems that the inhibition of AGEs and the oxidative stress built into the chemical structure of ARBs are what account for their renal benefits and not only blood pressure lowering and AT1 affinity [16,71]. 

Two novel orally active small molecules that inhibited plasminogen activator inhibitor (PAI) activity, TM5001 and TM 5007, were identified by Izuhara *et al.* [72]. Inhibition of PAI-1 may provide a novel treatment for obesity and anti-insulin resistance treatment [73]. Increased PAI-1 has been linked not only to thromobosis and firbrosis but also to obesity and insulin resistance [73]. Since PAI-1 is increased in kidneys of humans and animals with diabetic nephropathy and is association with extracellular matrix, its inhibition may be of great importance as a therapeutic tool in diabetic neprhropathy [74].

#### Natural Approach

Effect of dietary fiber mixture on metabolic syndrome was observed in this metabolic rat model. Abdominal obesity was attenuated and body weight was reduced compared to controls [75].

### 2.5. CRDH Rats

The construction of the Cohen Rosenthal Diabetic Hypertensive Rat (CRDH) model was initiated nearly a decade ago by cross-breeding between the Cohen Diabetic rat (CDR) and the SHR. The CDR is a rodent model that expresses the following phenotypes: when fed regular diet, it is non-diabetic; when fed a diabetogenic diet, it invariably develops diabetes reminiscent of type 2 in humans. The high-sucrose copper-poor diet should consist of 18% casein, 72% sucrose, 4.5% butter, 0.5% corn oil, 5% salt No. II USP, water, and fat-soluble vitamins. Thus, a diet very poor in copper is required to develop the full diabetic phenotype.

An outstanding feature of the CRDH rat is that it is a nonobese model of diabetes, which allows dissociation of the confounding obesity factor from other diabetogenic genes. Its pathology [76] includes severe diffuse diabetic glomerulosclerosis and severe hypertensive changes in arteries and arterioles, characterized by fibrinoid necrosis and/or "onion skin" lesions, as well as by smooth muscle cell hyperplasia. Such vascular changes were not observed in the CDR or in the SHR. Diffuse glomerulosclerosis with marked expansion of mesangial matrix as well as lipohyalin lesions were also occasionally observed. Myocardial changes are prominent, with foci of ischemic necrosis and hyperplastic vascular changes. CRDH rats undergo cardiac hypertrophy and vascular changes affecting small-sized coronary arteries. 

Oxidative stress values were higher in CRDH than are seen in SHR and CDR rats [77], as evaluated by the thermochemiluminiscence (TCL) analyzer (Lumitest Ltd., Nesher, Israel), an instrument that measures the susceptibility to oxidation of a test sample.

Studies performed with this model showed that omapatrilat, which inhibits both neuropeptidase (NEP) and ACE inhibitor, is a potent antihypertensive agent and has a beneficial effect on glycemic control, lowering glucose levels and improving insulin sensitivity [78].

Pharmacological treatment with lercanidipine (a calcium channel antagonist) in the CRDH rat countered left ventricle thickening and restored cardiocyte area values in subendocardium [79]. It also countered hypertension-diabetes-related cardiac and coronary changes, suggesting that this dihydropyridine-type calcium channel antagonist may improve heart and coronary structure in diabetes associated with hypertension.

Lercanidipine reduced systolic blood pressure and glucose level. While the small arteries and glomerular arterioles in the control group exhibited wall thickening and luminal narrowing, lercanidipine administration prevented changes in small-sized arteries and glomerular arterioles [80].

When the antihypertensive and hypoglycemic effects of telmisartan was studied in CRDH [80], blood pressure was significantly lowered by telmisartan in SHR and CRDH, independent of body weight, and remained fairly constant in controls throughout the experiment. Blood glucose levels fell rapidly only in the treated animals and remained steady in controls. Results indicate that telmisartan is a prototype of a new approach to treating coexisting diabetes and hypertension [81].

In a comparative study [82], telmisartan and valsartan (ARBs) significantly (P < 0.01) reduced blood pressure, while telmisartan and rosiglitazone, a PPAR-gamma agonist, considerably reduced blood glucose to normoglycemic levels. In contrast to valsartan, telmisartan significantly increased adiponectin (60%, P < 0.01) in CRDH, while rosiglitazone induced an increase in CRDH and CDR animals, less so with rosiglitazone. Telmisartan also induced downsizing of epididymal adipocytes compared to valsartan. The conclusion from this study is that in addition to its hypotensive effect, telmisartan demonstrated beneficial thiazolidinedione-like effects.

Another study [83] examined S-allylmercaptocaptopril (CPSSA), a conjugate of captopril with allicin, an active principle in garlic with multiple beneficial actions on metabolic-syndrome abnormalities. CPSSA integrated the anti-hypertensive feature of both allicin and captopril, making it a potential anti-diabetic and cardiovascular protective agent. No increase in weight was observed in contrast to control rats.

## 3. Discussion

The models discussed in this review are useful platforms on which basic science is translated into clinical medicine. They have been the vehicle for researching the classical factors contributing to the pathology of hypertension combined with diabetes –hypertension, hyperglycemia, hyperinsulinemia and hyperlipidemia – and have pointed to optimal treatments, although these treatments do not completely prevent renal complications. They are valuable for probing the mechanisms potentiating cardiovascular and renal morbid events in the setting of spontaneous hypertension and diabetes. The models have taught us the limitations of therapeutic agents and spurred the search for better modalities. Animal studies have pinpointed new culprits in the hypertension/diabetes picture that remain to be translated into clinical medicine, including hypoxia, oxidative stress, and advanced glycation. Several target molecules have been identified that need to be incorporated into a treatment modality.

One must remember that hypertension and diabetes may have the same phenotype in many patients, but their etiology and clinical course may differ significantly. For example, GK rats have sodium retention but they reach full-blown disease in the different animal models either by getting AII or dehydrocorticosteron or salt consumption. Thus, the parallels in humans must be drawn with great caution.

Treatment of metabolic syndrome means treating a multifactorial condition that is actually a conglomerate of health problems and therefore varies between individuals. Hence the further value of animal strains that represent all the different types of the syndrome. The challenge is to find suitable experimental models that not only replicate the various components of the syndrome, but have anomalies similar to those seen in metabolic syndrome patients. Such animal strains, in particular rat ones, will continue to be useful in assessing the efficacy of the drugs and lifestyle interventions we are seeking to treat or prevent the syndrome. Indeed, the strains described in this review display disorders very similar to those seen in the majority of patients with the disorder.

Clearly, the renal benefits of ARBs do not necessarily depend only on blood pressure lowering and AT1R affinity, but rather on the inhibition of AGEs and oxidative stress inherent to their chemical structure. R-147176 and other new sartan derivatives like TM2002 [70] open new avenues to the treatment of cardiovascular and kidney diseases. Altogether, renoprotection is not necessarily associated with blood pressure or glycemic control. In contrast, it is almost always associated with decreased AGE formation, which may reflect decreased oxidative stress as it is concomitant with a marked reduction in the markers of this parameter. Findings from research with ARBs point to the strong contribution of the RAS to the pathogenesis of renal damage in metabolic syndrome, and the powerful renoprotection conferred by ARBs to patients with metabolic syndrome [70]. There is experimental evidence that ARBs also decrease renal oxidative stress and advanced glycation, a fact that might help understand the link between ARBS and improved proteinuria. R-147176 was also found to ameliorate ischemic cerebral damage through anti-oxidative and anti-inflammatory properties; evaluation of the drug administered orally or intravenously reduced numbers of ED-1-positive cells and Tunel-positive cells, and protein carboxyl formation, in the damaged brain [84]

Cobalt chloride HIF activation did not correct hypertension and metabolic abnormalities, but reduced proteinuria as well as histological kidney injury. However, it is known to be toxic which can limit its use.

The nonpharmacological approach and the world of plants are also expanding our horizons enormously. Herbs alone and in combination have proven to be very effective and need to be incorporated into the armamentarium of the clinician. The hypoglycaemic and hypotensive properties of plant extracts have resulted in their use in traditional medicine for the treatment of diabetes. We have our plethora of animal models (Table 1). The issue now is to identify and interpret the clinical evidence in human trials.
